# Psychosocial Predictors of Body Weight Congruence in Adolescents Aged 15 and 17 Years in Poland: Findings from the Health Behaviour in School-Aged Children (HBSC) Study

**DOI:** 10.3390/ijerph19042342

**Published:** 2022-02-18

**Authors:** Anna Dzielska, Magdalena Woynarowska

**Affiliations:** 1 Department of Child and Adolescent Health, Institute of Mother and Child, Kasprzaka 17a Street, 01-211 Warsaw, Poland; 2 Department of Public Health, Faculty of Health Sciences, Medical University of Warsaw, Żwirki i Wigury 61 Street, 02-091 Warsaw, Poland; magdalena.woynarowska@wum.edu.pl

**Keywords:** adolescents, body weight congruence, psychosocial factors, body satisfaction, social comparisons, social media use, social support, social self-esteem

## Abstract

Background: Body weight congruence (BWC) has implications for adolescent health. The main goal of this study was to examine the distribution of BWC and its relationship with six psychosocial factors. Methods: A representative sample of N = 3508 adolescents aged 15 and 17 years (52.4% girls) derived from the Health Behaviour in School-aged Children study, conducted in 2017/2018 in Poland, was used. BWC groups were defined based on self-reported BMI and subjective assessment of weight: (1) correct perception; (2) overestimation, and (3). underestimation. Principal component analysis (PCA) extracted the following two factors: a socio-relational factor (SR) related to perceived social support and social self-efficacy, and a body attitudes and social media exposure factor (BAME). Using the total sample, multinomial logistic regression was applied to estimate their impact on the BWC, and gender-specific models were compared. Results: Half (48.6%) of the adolescents correctly estimated their body weight, 31.0% overestimated it (girls 43.9%, boys 17.1%), and 20.0% underestimated it (boys 37.2%, girls 9.0%). Overestimation of body weight concerns 48.0% of normal weight girls, 50.0% of underweight girls, and 21.3% and 32.1% of normal weight and underweight boys, respectively. The percentage of normal weight (34.4%), and overweight and obese (30.8%) boys who underestimated their body weight was three times higher than the respective percentages of girls that underestimated their weight (9.0% and 11.9%). The SR factor protected adolescents from both underestimation (only in girls) and overestimation in the total sample (OR 0.74, 95%CI 0.68–0.81) and both genders. BAME increased this risk of overestimation in both genders (OR = 1.83, 95%CI 1.67–2.0), and the risk of underestimation among boys. Conclusions: Prevention programmes should include a wide range of psychosocial factors to improve BWC among adolescents.

## 1. Introduction

Weight perception is a perceptual component of body image that relates to the assessment of body size [1]. Body weight congruence (BWC) refers to the correct perception of body weight in comparison with its actual value, while incongruence relates to overestimation or underestimation of one’s own body weight [2].

Research by Aloufi et al. shows that adolescents are a group particularly susceptible to inadequate weight perception, and the manifestation of this problem during adolescence may persist into adulthood [3]. According to the results of the Health Behaviour in School-aged Children (HBSC) study, girls are more likely than boys to consider themselves too fat, whereas boys are more likely than girls to report being too thin [4,5]. Approximately half of the girls who consider themselves too fat are those whose body weight-to-height ratio, according to self-reported BMI, is within the normal range [5]. Analysis based on HBSC data from 26 European countries shows that adequate weight perception occurs in approximately half of adolescents aged 11–15 years, and this percentage decreases with increasing age, especially among girls. Girls overestimate their body weight more than twice as often (26.4%) as boys (11.8%), and the percentage of overestimation is the highest among Polish girls compared to their counterparts in the analysed countries [6]. According to the international HBSC report, Polish adolescents also occupy unfavourable positions in international rankings in terms of perceived social support from parents and peers. In addition, a downward trend in the percentage of adolescents perceiving high social support has been observed [4,5]. Therefore, it seems reasonable to analyse BWC in Polish adolescents in the context of the potential influence of selected psychosocial factors. The data collected in Poland also include older age groups (17-year-olds) surveyed beyond the international protocol. This provides an opportunity to answer the question of how body weight congruence changes after the age of 15.

Inadequate weight perception, especially weight overestimation, is recognised as a key component of body image disturbance and is a known factor in the development of eating disorders [7], body dissatisfaction, social physique anxiety and low self-esteem [8], a less healthy diet and physical inactivity [9], problematic behaviours [10], poor mental well-being [11], and mental health disorders [12,13]. It can also have behavioural consequences, such as weight control intentions [14], problematic eating, unjustified dieting, and the use of other practices aimed at modifying body image [6,15,16,17].

Some studies show that underestimating one’s own excessive weight can be a serious public health problem, which is leading to a global increase in the prevalence of overweight and obesity and lowering the motivation to engage in healthy behaviours [18]. In addition, adolescents that underestimate their body weight are at risk of more frequent consumption of unhealthy snacks and fast food [19], higher risk of inappropriate weight control intentions, consumption of an unhealthy diet, and reduced physical activity behaviours [14], as well as poor well-being [11].

Adolescence is a key period in the development of body image, when numerous and intense changes take place in all areas of a child’s functioning [20]. These are initially changes of a physical nature, followed by affective-motivational and cognitive changes during psychological development, as well as changes in social development [21]. Given the developmental nature of this period of life, researchers have proposed multifactorial models involving biological and individual psychological characteristics, social influences, and interpersonal interactions with the aim of explaining the underlying factors that lead to, or protect against, the development of body image disturbances in adolescents [22,23]. To understand how adolescents’ problems with estimating their body size arise, it is important to look at social–cognitive processes, as well as the function of visual adaptation [3,24]. Although the distinction between body weight misperception and body dissatisfaction is emphasised [25,26], inadequate body weight perception may lead to body image disturbances. Congruence in weight assessment refers to the cognitive aspect of body perception, i.e., the ability to estimate body size correctly. Disturbances in this area are explained, among others, by the cognitive–behavioural model of body image development. It assumes that the problem with the perception of body image may be influenced by factors resulting from the individual’s experiences, the socialization process, as well as physical characteristics and personality attributes. This process influences the development of the body image schema. It can be activated by everyday events, for example, arising from social comparisons, but also the quality of the social relations and the readiness to initiate behaviour in social situations while making decisions to undertake specific behaviours [1]. It is also worth noting that some theoretical models, such as the Tripartite Model of Influence, point to three primary sources of influence (parents, peers, and the media) on the development of body dissatisfaction and disordered eating [27,28]. Meanwhile, among the factors influencing BWC, the most pronounced are also those related to adolescent physical self-perceptions [29], negative body perceptions and attitudes toward one’s own body internalization of societal standards of appearance through media exposure and social comparison mechanisms [30], or comments concerning the body or body weight from the adolescents’ proximate environment [31]. Some studies also emphasise the protective role of high social self-efficacy on body image perception [32]. Relatively few studies show the influence of social factors on the adequacy of body weight assessment in relation to its actual value [29,33]. Attention is drawn to the relationship between the socioeconomic situation of the family and the BWC [29], and a protective role of higher levels of social support received from parents and high-quality communication with parents against overestimating or underestimating body weight [33]. Considering the importance of the social context of development, it would also be interesting to examine the relationship of BWC to other psychosocial factors besides support from significant others. The research hypothesis establishes that there is inconsistency in the assessment of body weight by adolescents. Again, it was hypothesised that factors related to social relationships may protect adolescents from the risk of over- or underestimating body weight, while factors related to body dissatisfaction, greater propensity to make social comparisons regarding one’s own appearance, and the problematic use of social media may increase the risk of incongruent body weight assessment.

The aims of the study are as follows:

- To investigate the self-perception of body weight by adolescents aged 15 and 17 years in Poland, and to analyse its accuracy in terms of BMI, considering the possibilities of both overestimation and underestimation.
- To evaluate the relationship between BWC and selected psychosocial factors after correcting the analysis for age and gender

## 2. Materials and Methods

### 2.1. Surveyed Sample

The data from the Health Behaviour in School-aged Children (HBSC) survey were used. The HBSC survey is a cross-sectional survey conducted in collaboration with the WHO regional office. Data are collected in schools every four years, currently in 51 countries in Europe and North America. The HBSC protocol requires member countries to survey the following three age groups of school children: 11, 13, and 15 years of age. However, other age groups, especially older students, are allowed to be included in the survey. This procedure was used in Poland in the last round of the HBSC survey. The survey methodology, including sampling procedures, ethical requirements, and survey questions, were the same for the older age group as for the younger adolescents.

The presented survey was conducted during the 2017/18 school year on a representative sample of adolescents aged 15 and 17 years, students in the third year of junior high school and the second year of senior high school, respectively. The analyses included data from *N* = 3508 adolescents (52.4% girls) aged 15 (*N* = 1847) and 17 (*N* = 1661) from schools in all provinces of Poland, for whom no missing data were found for the key variables of body weight, height, and self-assessed body weight. The data collection procedure was conducted according to the standardised international HBSC protocol [34]. The basic sampling unit was schools. Detailed information on the HBSC survey methodology is presented in the national report and the international report of the HBSC survey [7,35].

### 2.2. Ethics

Approval from the Bioethics Committee on the data collection procedure and research tools was obtained (No. 17/2017, dated 30 March 2017). Participation in the study was anonymous and required the informed consent of the adolescents and their parents.

### 2.3. Variables and Indicators

#### 2.3.1. Body Weight Congruence

The main outcome variable was the congruence of subjective body weight assessment and body weight category according to BMI. The first question aimed to measure self-perception of body weight, as follows: ‘do you think your body is—“much too thin”, “a bit too thin”, “about the right size”, “a bit too fat”, or “much too fat”?’. The question was analysed in three categories, combining extreme answers. The variable was recoded in this way to reflected three body weight states—below normal weight, normal weight, and excess weight—which allowed us to combine it with the three BMI status categories reflecting the same body weight states, and finally, to calculate the body weight congruence.

Body weight categories according to BMI (underweight, normal weight, overweight and obesity) were calculated based on declarative data on the subjects’ body weight and height. Body weight was expressed in kilograms and body height in centimetres, both to one decimal place. For the calculation of BMI Z-score for sex and age (BMI-for-age), cut-off points were used according to the recommended reference values of the World Health Organization, 2007 [36,37]. Overweight and obesity were defined as BMI-for-age values >+1 SD and underweight as <−2 SD, separately for boys and girls.

Three categories of body weight congruence (BWC) were distinguished:

- Group 1: correct weight perception (BMI normal, think they are just right; BMI below normal, think they are too thin; with excess weight, think they are too fat).
- Group 2: overestimation of body weight (BMI normal, consider themselves too fat; BMI below normal, consider themselves just right or consider themselves too fat).
- Group 3: underestimation of body weight (BMI above normal, consider themselves too thin or just right; BMI normal, consider themselves too thin).

#### 2.3.2. Psychosocial Indicators of body Weight Congruence

##### Physical Appearance Comparison

We used the Polish version of the physical appearance comparison scale (PACS), which was included in the Polish HBSC questionnaire separate from HBSC international protocol, to evaluate the tendency to compare oneself with others. The version we used consisted of four items [30], adapted from the original five-item tool created by J.K. Thompson et al. [27,28]. The summary result of the scale was 16 points ranging from 0 to 16. Higher results indicated higher levels of social comparison of appearance. In the analysed sample of adolescents aged 15 and 17 years, four items make up a single component that explains 63% of the total variance. Moreover, the scale has a satisfactory level of internal consistency, as determined by a Cronbach’s alpha of 0.796 [38].

The variable was analysed as a continuous variable and also divided into three categories indicating the level of social comparisons of appearance, as follows: low (0–2 points), average (3–7 points), and high (8–16 points). 

##### Body Satisfaction

The body image subscale (BIS) was used to assess body satisfaction (BS). The scale was initially part of the body investment scale developed by Orbach and Mikulincer [39]. The scale consists of six items describing young people’s attitudes towards their own bodies. Adolescents rated the extent to which they agreed with the statements by selecting one of five answers, ranging from strongly disagree to strongly agree. Three items were reverse coded. The summary score of the scale ranged from 0 to 24 points, higher scores reflecting higher body dissatisfaction. In the analysed sample, six items make up a single component that explains 68.2% of the total variance. Moreover, the scale has a high level of internal consistency, as determined by a Cronbach’s alpha of 0.906 [38].

The variable was analysed as a continuous variable, as well as divided into the following three categories indicating the level of body satisfaction: high (0–3 points), average (4–11 points), and low (12–24 points). 

##### Problematic Social Media Use

Problematic social media use was defined by the diagnostic criteria, i.e., preoccupation, tolerance, withdrawal, persistence, escape, problems, deception, displacement, and conflict. The problematic social media use scale (PMSU) was measured with the original nine-item social media disorder scale (SMD) using a dichotomous (No(0)/Yes (1)) answer scale [40,41]. The summary score of the scale ranged from 0 to 9 points. Higher scores reflected more intensive problems with social media use. In the analysed sample, scale items make up a single component that explains 33.7% of the total variance. Moreover, the scale has a high level of internal consistency, as determined by a Cronbach’s alpha of 0.748 [38].

The scale was used as a continuous variable, as well as divided into three categories. The cut-off point for PSMU was set as 6-point scores or higher. Subsequent categories were defined as follows: no problems (0 points), some problems (1–5 points), problematic social media use (6–9 points). 

##### Social Self-Efficacy

Social self-efficacy (SSE) was measured using subscales of the Self-Efficacy Questionnaire for Children (SEQ-C) by Muris [42]. This scale includes eight items, which are rated on a 5-point scale from 0 (not at all) to 4 (very well). The item scores were summed, with scores ranging from 0 to 32 points. A higher score indicated higher student SSE. In the analysed sample, eight items make up a single component that explains 48.0% of the total variance. Moreover, the scale has a high level of internal consistency, as determined by a Cronbach’s alpha of 0.842 [38].

The scale was used as a continuous variable, as well as divided into three categories. There was no recommendation for specific cut-off points. The subsequent categories indicated the following levels of SSE: low (0–16 points), average (17–24 points), and high (25–32 points). 

##### Family Support

Family support (FS) was assessed on a four-item scale, which measured the perceived availability of emotional support and help within the family. The scale is one of three subscales that make up the multidimensional scale of perceived social support [43]. Students rated each of the four statements on a seven-point scale ranging from ‘very strongly disagree’ to ‘very strongly agree’. Summary score of the four items ranged from 0 to 24 points, where higher scores indicated higher levels of perceived FS. The four items make up a single factor that explains 85.3% of the total variance. The scale has high reliability, as indicated by the Cronbach’s alpha of 0.894 [38].

This scale was used as a continuous measure of family support, and also with a division of three categories indicating the level of perceived support, as follows: low (0–11 points), average (12–21 points), and high (22–24 points). 

##### Peer Support

Peer support (PS) was assessed by a four-item scale that measured the perceived availability of emotional support and help within the peer group. The scale, same as the scale for family support, is one of three subscales that make up the multidimensional scale of perceived social support [43]. Students rated each of the four statements on a seven-point scale ranging from ‘very strongly disagree’ to ‘very strongly agree’. Summary scores of the four items ranged from 0 to 24 points, where higher scores indicated higher levels of perceived PS. The overall score for the peer support scale was calculated by summing the scores for all items. This summary score ranged from 0 to 24, where higher mean scores indicated higher levels of perceived PS. The four items make up a single factor that explains 76.2% of the total variance. This scale has high reliability, as indicated by the Cronbach’s alpha of 0.941 [38].

The score was also categorised into the following three groups indicating the level of perceived PS: low (0–9 points), average (10–18 points), and high (19–24 points). 

### 2.4. Methods of Data Analysis

Sample characteristics were presented by descriptive statistics using the χ^2^ test. The body weight congruence was computed. Moreover, six psychosocial factors were used as continuous variables or presented as three categories according to the recommended cut-offs, or alternatively, if no recommendations were available, distributed on a discretionary basis so that approximately half of the responses fall in the middle range.

Multinomial logistic regression models were estimated in which the dependent variable was nominal and characterised by three values corresponding to overestimation, underestimation, and accurate weight perception. The latter category was the reference point. Using principal component analysis (PCA), we identified factors based on the six continuous individual and social variables mentioned above. The factors reflected two groups of potential influence on BWC, which were:

- The socio-relational factor (SR), comprising SSE, FS, and PS. The three scales explained 29.1% of the variance, with factor loadings ranging from 0.580 to 0.821. The Cronbach’s alfa was 0.611.
- The body attitudes and exposure to media messages factor (BAME), comprising PACS, BS, and PSMU. The three scales explained 27.7% of the variance, with factor loadings ranging from 0.633 to 0.806. The Cronbach’s alfa was 0.610.

The extracted factors were presented as Z-scores. For the total sample, the mean value was equal to 0, and the SD was 1. In this form, the factors were included in the model as covariates. Models were adjusted for age and gender.

In all analyses, *p* < 0.05 was considered to indicate statistical significance. Analyses were performed using the IMB package SPSS v. 27. WHO AnthroPlus software, which is intended for the global application of the WHO 2007 reference index for 5–19-year-olds to monitor the development of school-age children and adolescents. This software was used to calculate BMI categories [44].

## 3. Results

### 3.1. Body Weight Perception, BMI Categories, and Body Weight Congruence

More than 40.0% of students considered themselves too fat, and less than one in five assessed their body weight as too low. Self-perception of being too fat was statistically significantly more frequent among girls, and of being too thin, among boys. Self-assessment of body weight did not differ by age. Overweight or obesity occurred in 14.8% of adolescents, more than twice as frequently in boys than in girls. Underweight was significantly more frequent in 15-year-old adolescents than in 17-year-olds (Table 1).

Approximately half of the adolescents correctly assessed their body weight in relation to the self-reported BMI category. Overestimation of body weight was more than twice as frequent in girls than in boys, and the percentage of under estimators was more than three times higher in boys than in girls. Body weight congruence did not differ significantly by age (Table 1).

**Figure 1:**
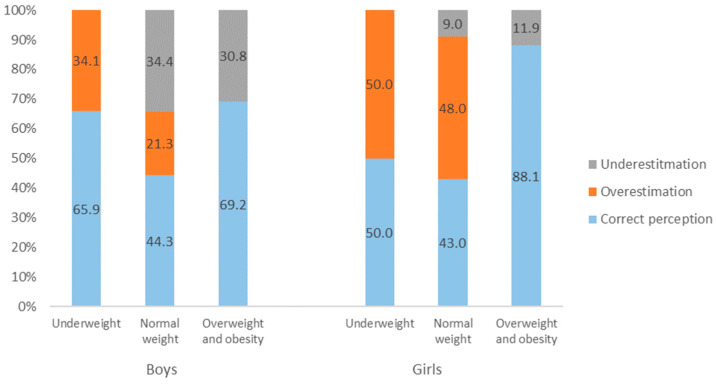
Body weight congruence for BMI categories.

Adequate weight assessment was most common in girls and boys who were overweight based on self-reported BMI, and was the least frequent in students of normal weight. Approximately half of girls and one quarter of boys classified as normal weight overestimated their weight. Half of girls and one in three underweight boys also overestimated their body weight. Body weight underestimation was almost three times more frequent in boys than in girls classified as overweight, and more than three times more frequent in boys than in girls within the normal range according to BMI (Figure 1).

### 3.2. Body Weight Congruence for Individual and Social Factors

Table 2 presents the mean values for the six continuous variables relating psychosocial factors potentially related to body weight congruence to the frequency distribution of the categorised variables, and the level of analysed factors in the total sample.

Statistically significant gender differences were observed in five out of the six factors examined, and negative outcomes were more common among girls. Girls were more likely than boys to have an increased tendency (high level) to compare themselves with others (35.7% vs. 17.0%, *p* < 0.001), to present a high level of body dissatisfaction (36.8% vs. 19.0%, *p* < 0.001), and to exhibit more problematic use of social media (10.8% vs. 6.8%, *p* < 0.001). Moreover, girls were less likely to feel high levels of support from family (25.3% vs. 28.9%, *p* < 0.001). However, feeling high levels of support from peers was more common in girls than in boys (32.1% vs. 26.1%, *p* < 0.001). No gender differences were noted in terms of SSE. Age-related differences were noted for social media use, with a higher prevalence of problematic use among younger (10.2%) than among older (7.2%) adolescents (*p* = 0.004), as well as for peer support, of which a high level was more often experienced by older (31.4%) than younger (25.0%) students (*p* < 0.001).

Results of the analyses indicated a statistically significant association of body weight congruence with all individual and social factors (Table 3). The percentage of adolescents who perceived their weight correctly was highest among those with a low tendency to compare their appearance with others, a high satisfaction with body image, no problems with social media use, a high level of social self-efficacy, and a high level of family as well as peer support; correct perception decreased with deterioration in the factors analysed. Similar patterns of change in the percentages according to the variables analysed were noted for the underestimation of body weight, except for the relationship with peer support. In this case, the relationship was non-linear, with the highest percentage of students that underestimated their body weight occurring in the group with average levels of peer support, and the lowest in adolescents with high levels of support.

The opposite applied to the category of overestimation of body weight. The groups of adolescents with the highest tendency towards social comparisons of appearance, the worst body image, problematic social media use, the lowest social self-efficacy, and the lowest level of family and peer support, constituted the highest percentage of adolescents that overestimated their body weight; this proportion decreased as the factors analysed improved.

### 3.3. Psychosocial Predictors of Body Weight Congruence in Multinomial Regression Models

Three multinomial logistic regression models were estimated in which the dependent variable was daily weight congruence in three categories and the independent variables were two groups of factors reflecting SR and BAME standardised indices. The first model was adjusted for gender and age, then the two gender-specific models were compared (Table 4).

Pearson’s χ^2^ test indicated that the model fits the data well (χ² (6478) = 6504.990, *p* = 0.404). Furthermore, the deviance chi-square indicated a good fit (χ² (6478) = 6044.295, *p* = 1.00). The value of the Nagelkerke pseudo R-square was 0.216.

The Parameters in the constant model testing the factors influencing the risk of the overestimation of body weight in comparison to congruent perception of body weight were: b = −1.506, s.e. = 0.662, *p* = 0.023. The risk of overestimation of body weigh decreased significantly with a higher Z-score of the socio-relational dimension (b = −0.297, s.e. = 0.043, *p* < 0.001). Furthermore, the risk of overestimation increased with higher Z-sores of the appearance comparison and the social media exposure-related dimension (b = 0.603, s.e. = 0.046, *p* < 0.001). Additionally, girls were more likely to overestimate their weight than boys (b = 0.690, s.e. = 0.094, *p* < 0.001).

In the general model, none of the main factors were related to the underestimation of body weight, with the exception of gender. The parameters in the constant model were: b = −0.803, s.e. = 0.739, *p* = 0.278. Girls were less likely than boys to underestimate their weight (b = −1.306, s.e. = 0.111, *p* < 0.001).

Both models that estimated separately by gender had good-fitting parameters, although the fit indices were notably better in the girls’ model. The Pearson’s χ^2^ test result was χ² (3056) = 3051.787, *p* = 0.518 for the boys’ model and χ² (3416) = 3474.858, *p* = 0.237 for the girls’ model, and the deviance chi-square results were χ² (3056) = 3052.110, *p* = 0.516 and χ² (3416) = 2967.310, *p* = 1.000, respectively. However, the value of the Nagelkerke pseudo R square was higher in the girls’ (0.135) than the boys’ (0.041) model.

As observed in the overall model, both factors from the SR and the BAME groups proved to be significant predictors of overestimation in boys (b = −0.316, s.e. = 0.072, *p* < 0.001; b = 0.454, s.e. = 0.083, *p* < 0.001, respectively) and girls (b = −0.303, s.e. = 0.055, *p* < 0.001; b = 0.659, s.e. = 0.057, *p* < 0.001, respectively). However, the analysis revealed different predictors of underestimation of body weight for boys and girls. In the boys’ model, factors related to BAME increase the risk of underestimating body weight significantly (b = 0.158, s.e. = 0.069, *p* < 0.001). An important predictor of underestimation in girls, that reduced the risk of underestimating body weight, was a higher Z-score of the SR dimension (b = −0.300, s.e. = 0.090, *p* < 0.001).

## 4. Discussion

The purpose of this study was to investigate body weight congruence in adolescents based on their perceived weight as too thin, about right, or too fat, as well as three categories of nutritional status based on BMI (underweight, normal weight, overweight and obesity). Moreover, the associations between BWC and the selected psychosocial factors, including body image satisfaction, age, and gender, were examined. The analysis was performed on a representative sample of 3508 adolescents aged 15 and 17 years.

The results revealed that approximately half (48.6%) of the adolescents aged 15 and 17 years correctly estimated their body weight, with similar proportions for girls and boys. Every third teenager overestimated their body weight, whereas every fifth underestimated it. Overestimation was more prevalent in girls than boys; conversely, underestimation was more frequent in boys than girls. The results are in line with other studies conducted among adolescents [2,3,7,29,45,46,47].

Our study provided an opportunity to compare the BWC between groups of 15 and 17-year-olds. The prevalence of adequate body weight assessment and the two directions of its discrepancy were not significantly different between these groups. Previous analyses among 11- to 15-year-olds from 26 European countries show that weight perception deteriorates with age during this period of life [6]. Analyses conducted by Ben-Yaish et al. among Israeli adolescents indicated differences in the adequacy of body weight perception between younger (aged approximately 11–14 years) and older students (aged 15–18 years). A comparison showed significantly higher proportions of adolescents with inadequate weight perception in older age groups [33]. Our analyses would suggest that this process among Polish adolescents slows down after the age of 15, and stabilises in the subsequent years, whereas inadequate weight assessment is less common in younger children [6,48,49]. Ultimately, an interesting direction for future analyses of Polish adolescents would be to compare changes in BWC for an extended age group, both in older adolescents and in young adults, as well as in younger children, to determine which stages in life do significant changes in BWC occur, while simultaneously providing support toward targeting prevention efforts at specific areas of child and adolescent functioning within certain age groups.

An analysis of the association of BWC with nutritional status based on BMI in a sample of Polish adolescents shows that, in the group of adolescents with normal body weight, underestimation of weight is more than three times more frequent in boys, and overestimation is twice as frequent in girls. Our results also show that most overweight adolescents are aware that they weigh too much, although this correct weight perception is less common in boys. Failure to perceive an overweight problem is three times more prevalent among boys than among girls. Similar results indicating that 34.0% (95% CI 25.0%–43.0%) of children tended to underestimate their overweightness were observed in a meta-analysis of 91 papers published by Alshahrani et al. in 2021 [50]. One explanation for the greater tendency for boys, especially those that are overweight or obese, to underestimate their body weight may be the mechanism described in the ‘visual normalization’ theory [51]. This theory suggests that underestimation of body weight, especially underestimation of overweight and obesity, may be due to visualization norms that are formed by intense exposure to a popular, frequently occurring silhouette pattern in the environment [51,52,53]. According to our study, underestimation of body weight was significantly more common in boys. A higher percentage of overweight and obese adolescents, according to their self-reported nutritional status, was also noted in this group, which may have contributed to a greater acceptance of greater body weight and, consequently, its underestimation.

The data presented in this paper clearly demonstrate that a fairly high proportion (57.4%) of underweight adolescents overestimated their weight. Even higher percentages were observed by Lotrean et al. among younger adolescents (aged 11–14 years) from Romania, where up to 61.5% of children who were underweight considered their weight to be normal [54]. While the results among underweight girls indicating that half of them overestimated their body weight were expected (50.0%), it is worth noting that nearly one-third (31.0%) of underweight boys also perceived their body weight to be greater than their actual weight. The results of a French study conducted among adolescents of a similar age (9th grade) indicated the same proportions in both genders as in our study. The percentages of boys (thinness grade I 47.0% and grade II 81.0%) and girls (thinness grade I 89.0% and grade II 62.0%) overestimating their weight despite being objectively underweight were, according to these findings, even higher than those in our sample [55].

Our analyses also focused on exploring the association of selected psychosocial factors with BWC. We limited the factors that may influence BWC to gender, age, BMI category, body satisfaction, social support from peers and parents, physical appearance social comparisons, and self-esteem in social situations, as well as social media abuse. There are some literature reports of the influence of some of these factors on weight assessment compliance. However, the available data either only consider selected factors or do not include older adolescents. In order to avoid multicollinearity resulting in less reliable statistical interference, two factors obtained by the PCA method were included in the regression model instead of the six primary variables. A similar approach has been used in other studies based on HBSC results, where overall indices of social environment or physical activity assessment were proposed [33,56]. The PCA conducted in this study suggests that variables related to social relationships (peer support, family support, and social self-efficacy) form one factor (SR) and the other three (physical appearance social comparisons, body satisfaction, and problematic social media use) form another factor (BAME).

It is worth noting that we found common variability in the PACS, BS, and PSMU. The association between the BS and PACS seems to be clear, and the association of these two factors with social media use has been confirmed in other studies. These factors appear to have a stronger psychological background [57]. Although scientific evidence of direct effects of social comparisons of appearance on BWC is limited, the impact of such comparisons on the risk of body dissatisfaction is well demonstrated, showing that appearance-related social comparison induces individuals to experience depressing feelings, such as body dissatisfaction [58]. In turn, satisfaction with one’s own body is related to adolescents’ inadequate weight assessment [8,59]. At the same time, for social comparisons of appearance to have an impact on self-perceptions of appearance, they ought to interact with the feedback regarding one’s appearance that the adolescent receives from the proximate environment or the appearance patterns that one observes and internalises, for example through exposure to social media [60,61].

Our results indicate the protective role of the SR factor in overestimation of body weight among boys and girls, with a stronger positive impact for girls. This factor also protected girls from underestimation of their body weight. Studies by Ben-Yaish et al. [34] also showed the protective impact of social support. The BAME factor was found as a risk factor of overestimation in both boys and girls, and additionally increased the risk of underestimation among boys. This result shows that the interrelationship of increasing social comparisons of appearance, problematic social media use, and body dissatisfaction may have a negative impact, not only on eating disorders as mentioned in previous studies [62,63], but also on body weight congruence. The link between inadequate weight assessment and several adverse health consequences for adolescents is well recognised. It is therefore essential that efforts are made to identify the factors that should be acted upon in educational and/or intervention programmes targeting adolescents to reduce the risk of discrepancies in this assessment. Our results provide useful insights for practical action by indicating the need to simultaneously influence a group of factors related to cognitive function, behaviour, social relationships, and social self-efficacy in educational programmes.

### Strengths and Limitations

The strength of this study lies in the large representative sample, which captured the perspectives of both genders and permitted the analysis of both overestimation and underestimation of body weight. On the methodological side, an interesting approach was the use of multinomial logistic regression, which allowed the use of a dependent variable taking on three values.

However, our study has several limitations that are partly due to the data source chosen. The first limitation is the cross-sectional nature of the HBSC survey, which allows the description of relationships between variables in a random sample from the population, but limits the possibility of inferring a causal relationship between the studied variables. Secondly, in the set of variables presented in the analysis, there is no reference to puberty, particularly the pubertal timing, which may be an important factor influencing BWC formation during adolescence [22]. Despite this potential importance, in the 2018 round of the HBSC survey in Poland, no such questions regarding puberty were asked of young people. Thirdly, the HBSC questionnaire relies on self-reported height and weight data, which are prone to error. Several studies have shown that measured data correlate highly with self-reported data [64,65,66,67]. However, the level of discrepancy may depend on overall satisfaction with one’s body [68]. If the research had been planned for this topic from the beginning, the use of other questions could have been considered. For example, the problematic media use scale correlates with time spent online and attention to content acquired, with no direct reference to body image. In the future, items regarding posting your own photos, looking at celebrity photos, or interest in specific sites that influence body image, would be worthwhile.

## 5. Conclusions

Approximately half of Polish adolescents aged 15 and 17 years correctly assessed their body weight in relation to objective indicators based on self-reported BMI. Girls overestimated their body weight more often than boys, whereas boys underestimated their body weight more often than girls. The lack of an age-related difference in BWC may indicate a stabilization of this assessment in older adolescents, albeit at an unfavourably high level. Socio-relational factors protect adolescents from body weight overestimation, while factors related to negative body attitudes and problematic social media exposure increase this risk. The obtained results indicate the need to include prevention programmes for older adolescents in Poland, work on a wide range of psychosocial factors, strengthen their social skills, support them in building positive social relations, and enhance their individual potential. Further research is needed to explore and understand the age differences in younger and older children, and to examine the broader context of weight congruence formation in adolescents so that appropriate interventions may be designed for different age groups.

## Figures and Tables

**Table 1:** Total body weight perception, BMI categories, and body weight congruence, organised by gender and age.

	Total *N* %	Boys *N* %	Girls *N* %	15-Year-Olds *N* %	17-Year-Olds *N* %
Body weight perception					
too thin	653	479	174	354	299
	18.6	28.7	9.4	19.2	18.0
about right	1409	671	738	735	674
	40.2	40.2	40.2	39.8	40.6
too fat	1446	521	925	758	688
	41.2	31.1	50.4	41.0	41.4
χ^2^, *p*		251.225, <0.001		0.802, =0.670	
BMI category					
underweight	94	44	50	67	27
	2.7	2.6	2.7	3.6	1.6
normal weight	2895	1267	1628	1501	1394
	82.5	75.8	88.6	81.3	83.9
overweight or obese	519	360	159	279	240
	14.8	21.6	8.7	15.1	14.5
χ^2^, *p*		14.084, <0.001		115.647, <0.001	
Body weight congruence					
correct perception	1705	839	866	908	797
	48.6	50.2	47.1	49.1	48.0
overestimation	1091	285	806	568	523
	31.1	17.1	43.9	30.8	31.5
underestimation	712	547	165	371	341
	20.3	32.7	9.0	20.1	20.5
χ^2^, *p*		447.324, <0.001		0.486, =0.784	

**Table 2:** Descriptive statistics of categorical and continuous variables of six psychosocial factors.

	The Level of the Variable				
	Low	Average	High		
	*N* %	*N* %	*N* %	M SD	Cronbach’s Alpha
Appearance comparison (PACS)	911	1634	963	5.31	0.796
	26.1	46.9	27.0	3.72	
Body satisfaction (BS)	1043	1688	777	8.30	0.906
	28.4	49.0	22.6	5.67	
Problematic social media use (PSMU) ^1^	1200	1351	248	1.94	0.748
	42.9	48.3	8.9	2.11	
Social self-efficacy (SSE)	821	1633	970	20.74	0.842
	24.0	47.7	28.3	6.04	
Perceived family support (FS)	843	1702	942	16.14	0.894
	24.2	48.8	27.0	6.59	
Perceived peer support (PS)	863	1651	981	14.14	0.941
	24.7	47.2	28.1	6.39	

^1^ PSMU categories: no problems, some problems, problematic use.

**Table 3:** Body weight congruence for six psychosocial factors (%).

	Body Weight Congruence			
	Correct Perception	Overestimation	Underestimation	χ^2^, *p*
Appearance comparison (PACS)				
Low level	57.0	20.0	23.1	
Average level	48.0	30.0	22.0	127.605, <0.001
High level	42.2	43.6	14.3	
Body satisfaction (BS)				
High	65.1	8.9	26.0	
Average	50.7	27.7	21.6	448.182, <0.001
Low	32.0	55.0	13.0	
Problematic social media use (PSMU)				
No problems	54.2	24.2	21.7	
Some problems	45.8	34.0	20.1	55.066, <0.001
Problematic use	39.5	44.8	15.7	
Social self-efficacy (SSE)				
Low level	45.3	34.2	20.5	
Average level	47.9	32.0	20.1	12.148, =0.016
High level	52.7	27.6	19.7	
Perceived family support (FS)				
Low	40.2	42.0	17.8	
Average	48.7	30.5	20.8	79.490, <0.001
High	55.9	22.7	21.3	
Perceived peer support (PS)				
Low	45.0	35.1	19.9	
Average	48.1	29.4	22.5	20.255, <0.001
High	52.3	30.6	17.1	

**Table 4:** Risk of body weight congruence for two groups of psychosocial factors.

	Body Weight Congruence ^1^	
	Overestimation	Underestimation
Independent Variables	OR (95%CI)	OR (95%CI)
Total group		
Gender		
Girl	1.997 (1.661–2.400)	0.271 (0.218–0.337)
Boy (ref.)		
Age	1.034 (0.957–1.118)	1.025 (0.940–1.119)
SR ^2^ (Z-score)	0.743 (0.683–0.809)	0.932 (0.847–1.026))
BAME ^3^ (Z-score)	1.828 (1.669–2.002)	1.113 (0.997–1.243)
Boys		
Age	1.110 (0.974–1.267)	1.075 (0.968–1.194)
SR (Z-score)	0.729 (0.633–0.840)	1.013 (0.902–1.137)
BAME (Z-score)	1.574 (1.339–1.851)	1.171 (1.022–1.342)
Girls		
Age	0.993 (0.901–1.095)	0.915 (0.777–1.078)
SR (Z-score)	0.739 (0.664–0.822)	0.741 (0.621–0.884)
BAME (Z-score)	1.933 (1.727–2.163)	0.926 (0.761–1.127)

^1^ Congruence (ref.); ^2^ SR, socio-relational; ^3^ BAME, body attitudes and social media exposure.

## Data Availability

The data presented in this study are available on request from the corresponding author. The data are not publicly available due to internal HBSC data access policy. Data access to previous HBSC rounds is provided by the HBSC Data Management Centre—Department of Health Promotion and Development, University of Bergen (https://www.uib.no/en/hbscdata) (accessed on 10 December 2021).
